# Liver Resection for Gastroenteropancreatic Neuroendocrine Tumors with Extrahepatic Disease

**DOI:** 10.3390/jcm13174983

**Published:** 2024-08-23

**Authors:** Kelly M. Mahuron, Kristen E. Limbach, Matthew C. Hernandez, Philip H. G. Ituarte, Daneng Li, Jonathan Kessler, Gagandeep Singh

**Affiliations:** 1 Department of Surgical Oncology, City of Hope National Medical Center, Duarte, CA 91010, USA; kmahuron@coh.org (K.M.M.);; 2 Department of Medical Oncology, City of Hope National Medical Center, Duarte, CA 91010, USA; 3 Department of Radiology, City of Hope National Medical Center, Duarte, CA 91010, USA

**Keywords:** neuroendocrine tumors, liver metastases, liver-direct therapy, extrahepatic disease

## Abstract

**Background**: Although survival outcomes for neuroendocrine liver metastases (NETLM) are improved with liver-direct therapies (LDT), including hepatic debulking and nonsurgical trans-arterial embolization, the benefit is less established in the setting of concurrent extrahepatic disease (EHD). We performed a population-based study to characterize the rates of LDT being performed for NETLM with EHD patients and whether LDT is associated with survival outcomes. **Methods**: Patients with NETLM and EHD were identified using the California Cancer Registry database merged with data from the California Office of Statewide Health Planning and Development between 2000 and 2012. Demographics, clinical characteristics, and survival outcomes were analyzed for these patients with and without LDT. **Results**: 327 NETLM patients with EHD were identified. EHD sites included lung, peritoneum, bone, and brain. A total of 71 (22%) of these patients underwent LDT. Compared to NETLM with EHD patients who did not undergo LDT, patients who received LDT had longer median overall survival (27 vs. 16 months, *p* = 0.006). Within the LDT group, 23 patients underwent liver resection. Liver resection was associated with longer median overall survival compared to nonsurgical LDT (138 vs. 13 months, *p* < 0.001). **Conclusions**: LDT candidacy should be determined for patients on a case-by-case basis, but the presence of EHD should not preclude LDT with appropriate patient selection.

## 1. Introduction

Gastroenteropancreatic neuroendocrine tumors (GEP-NETs) are a heterogeneous group of tumors typically classified by their site of origin, whether they secrete functional hormones, and their state of cellular differentiation [1]. Although GEP-NETs are considered rare tumors, they have had a rising incidence and prevalence over the past 40 years [2]. While the majority of GEP-NETs demonstrate an indolent behavior, liver metastases are frequent and occur in up to 75% of patients [3]. For appropriately selected GEP-NET patients with liver metastases, hepatic debulking has been correlated with improved survival [4].

In addition to liver metastases, a significant proportion of GEP-NET patients also have extrahepatic disease (EHD) [5]. Although the presence of EHD has been associated with a worse overall survival [6], hepatic tumor burden is still often the main driver of mortality. Therefore, the benefit of hepatic cytoreduction in the setting of limited extrahepatic disease is unknown [7]. While EHD was once felt to be an absolute contraindication for liver debulking, consensus guidelines now acknowledge that the decision to perform hepatic cytoreduction is multifactorial and the presence of EHD should not necessarily preclude it [8,9].

As an alternative to liver resection, liver-directed therapies (LDT) can be performed in patients with extensive or progressive liver disease not amenable to resection or in patients that are poor surgical candidates. Two commonly utilized techniques include trans-arterial chemoembolization (TACE) with gel foam combined with chemotherapy or drug-eluting beads and trans-arterial radioembolization with yttrium-90 (Y90) [10]. These trans-arterial embolization procedures capitalize on the principle that NETLMs derive their blood supply nearly exclusively from the hepatic arterial system while liver parenchyma relies on portal venous inflow. Therefore, tumors can be selectively targeted without damaging normal liver. Both approaches have been associated with significant symptom control and tumor response [11,12].

In this population-based study, a California database was used to identify GEP-NET patients with concurrent liver metastases and EHD who underwent liver resection or LDT (TACE and Y90). Along with characterizing treatment patterns for these patients, we aimed to determine whether hepatic debulking was correlated with improved survival in the setting of EHD.

## 2. Materials and Methods

### 2.1. Data

Patient data from the California Cancer Registry (CCR) was merged with California Office of Statewide Health Planning and Development (OSHPD) discharge data to create a population-based, retrospective study design of patients with neuroendocrine neoplasms. The CCR is a statewide cancer registry that reports demographic, clinical characteristics, treatment, and survival information. As reporting for cancer care is mandatory in the state of California, it is one of the most comprehensive cancer registries in the United States.

The OSHPD database contains admission and discharge information from all general acute care, nonfederal facilities in California. Inpatient and outpatient care settings are included. Diagnoses and procedures for each record are coded according to the International Classification of Disease 9th Edition (ICD-9-CM). For this study, CCR–OSHPD linked records from 2000–2012 were used to identify records of patients with neuroendocrine neoplasms metastatic to the liver and with additional sites of extrahepatic metastases. The study was conducted in accordance with the Declaration of Helsinki, and human subjects review and approval for use of the CCR–OSHPD linked data were obtained from institutional and state-level institutional review boards. Patient consent was waived as this study was conducted using data from a de-identified database.

### 2.2. Patients

Patients were identified according to ICD-0-3 histology code for neuroendocrine neoplasms (NENs) (8240–8246, 8249, and 8150–8152). Site-specific codes were also used to identify NEN neoplasms of specific origins: gastrointestinal (C160–C166, C168–C169), pancreas (C250–C254, C257–C259), small intestine (C170–C173, C178–C179), and colorectal, including appendix (C180–C189, C199, C209). Our study included patients older than 18 years old with histologically confirmed NENs who had a diagnosis of liver metastases determined by the ICD-9 diagnosis codes of 197.7 (malignant neoplasm of liver, secondary) and 209.72 (secondary NET of liver). Sites of extrahepatic disease (EHD) included the lung (ICD-9 197.0), peritoneum (ICD-9 197.6), bone (ICD-9 198.5), and brain (ICD-9 198.3). Patients were determined to have concurrent EHD if the dates of the EHD were prior to or on the date of diagnosis of liver metastases. Patients with neuroendocrine carcinomas (NEC), without histologic confirmation of disease, with an unknown or more than one primary tumor site were excluded.

### 2.3. Data Variables

The following variables were collected for analysis: age, sex, race/ethnicity, comorbidities based upon the Charlson–Deyo score [13], primary NET tumor site, site of EHD, whether the primary tumor was resected, the receipt of liver-direct therapies (liver resection (with or without ablation), trans-arterial chemoembolization (TACE) and yttrium-90 radioembolization (Y90)), and receipt of chemotherapy.

### 2.4. Statistical Analysis

The primary outcome of this study was overall survival (OS). Student’s *t* test was used to compare continuous variables between groups. The chi-square test was used for categorical variables. Survival curves were compared using the Kaplan–Meier method and the log-rank test. Follow-up was defined as the time from date of diagnosis to the date of death or last contact. For OS, a failure event was defined as death from any cause. Patients alive at last follow-up were censored. Univariate analysis was performed using the Cox proportional hazards model. Statistical significance was set at *p* < 0.05 with assumption of two-sided tests. All statistical analysis was performed using Stata MP 14.2 software (StataCorp LLC, College Station, TX, USA).

## 3. Results

A total of 9728 patients with GEP-NETs were identified. In this study, patients with poorly differentiated histologic grade consistent with neuroendocrine carcinomas (NECs) were excluded as these patients have highly aggressive disease with poor survival [14]. Primary tumor sites included rectal (41%), small bowel (21%), pancreas (18%), gastric (11%), and colon (9%). A total of 1434 (15%) of GEP-NET patients had liver metastases (NETLM) and the frequency of patients with LM varied by primary tumor site (3% for rectal, 7% for gastric, 15% for small bowel, 25% for colon, and 40% for pancreas). In the setting of NETLM, rates of primary NET resection, liver resection, and TACE/Y90 also varied by primary tumor site (Table 1). Small bowel NET had the highest rate of primary NET resection, while pancreatic NET had the lowest (61% vs. 9%). Small bowel NETs had the highest frequency of liver resection (22%) and rectal NETs had the highest frequency of LDT (32%). As shown in Table 1, median overall survival (OS) also differed by primary NET site and whether LM were present.

**Figure 1:**
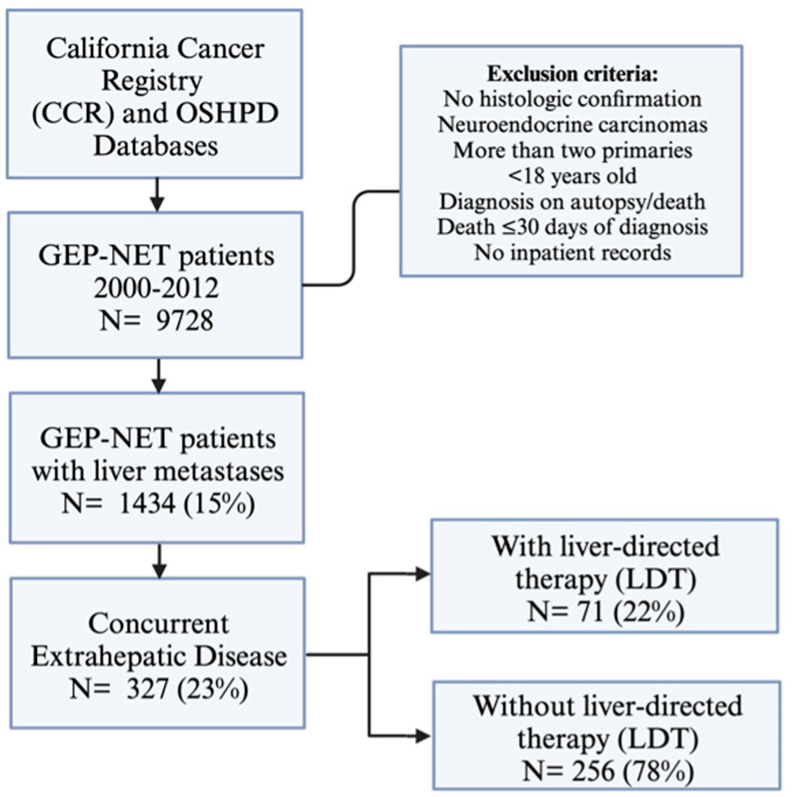
Study design and inclusion/exclusion criteria.

A proportion of 23% (327/1434) of NETLM patients had concurrent EHD. EHD sites included the peritoneum (49%), bone (37%), and lung (28%). Only 71 (22%) of NETLM with EHD patients received some form of liver-directed therapy (LDT), which included liver resection/ablation, TACE, and Y90 (Figure 1).

Patient demographics and clinical characteristics are shown in Table 2. NETLM patients with EHD who underwent LDT were younger (*p* < 0.001) but similar in sex, race/ethnicity, and comorbidities. The groups had comparable distributions of primary NET sites and rates of primary NET resection.

Regarding patterns of EHD, patients who underwent LDT had a higher frequency of concurrent peritoneal metastases (PM) (64% vs. 44%, *p* = 0.002), and lower frequencies of lung metastases compared to the no LDT group (18% vs. 30%, *p* = 0.043). The proportion of patients with multiple sites of EHD was similar between the LDT and no LDT groups. The group undergoing LDT had a higher percentage of patients who received chemotherapy at any time in their treatment course compared to the no LDT group (54% vs. 35%, *p* < 0.001) (Table 2).

**Figure 2:**
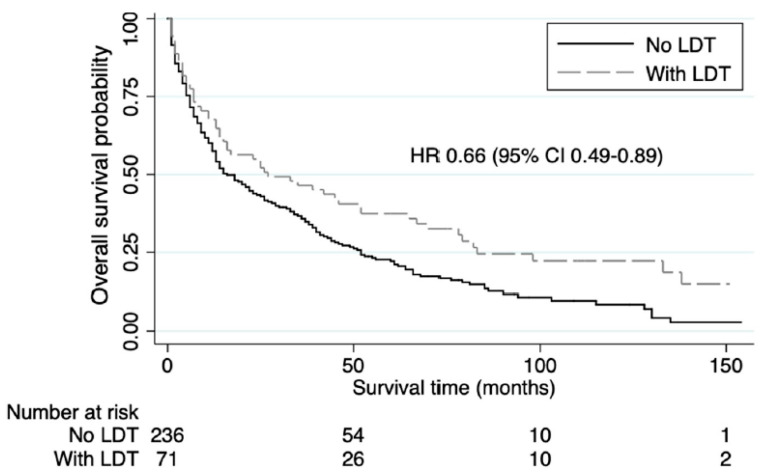
Kaplan-Meier Survival analysis showing overall survival in patients with gastroenteropancreatic neuroendocrine liver metastases and concurrent extrahepatic disease stratified by whether they underwent liver-directed therapy (LDT).

The 5-year OS rate for NETLM with EHD patients with LDT was 37% compared to 22% for the no LDT group. LDT was associated with significantly increased median OS (mOS) (27 vs. 16 months, HR 0.66 (95% CI 0.49–0.89), *p* = 0.006) (Table 2, Figure 2).

**Figure 3:**
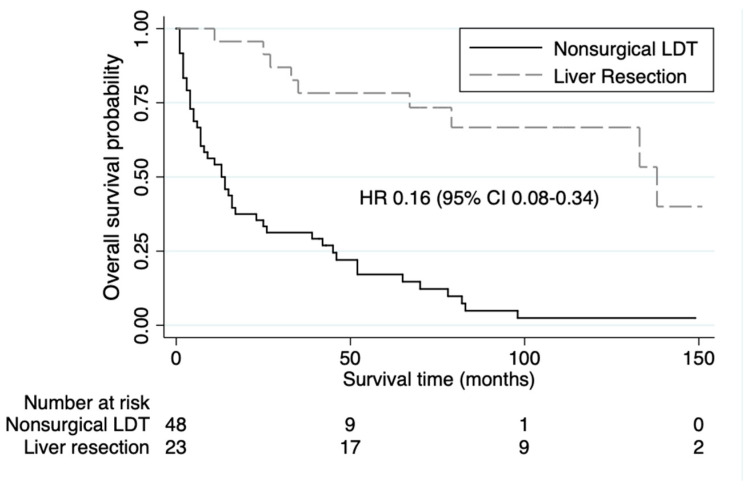
Kaplan-Meier Survival analyses showing overall survival in patients with gastroenteropancreatic neuroendocrine liver metastases and concurrent extrahepatic disease stratified as to whether they underwent nonsurgical liver-direct therapy (LDT) or liver resection.

While surgical resection or debulking is the preferred treatment for NETLMs, nonsurgical LDT may be performed due to poor patient fitness or tumor burden not amenable to resection [15]. Therefore, GEP-NET patients who underwent surgery may have different outcomes than those with nonsurgical approaches. In this study, 23 (7%) of the 327 NETLM and EHD patients underwent surgical resection and had a 5-year OS rate of 78%. Compared to the nonsurgical LDT patients, NETLM and EHD patients who underwent liver resection had a longer mOS (138 vs. 13 months, HR 0.16 (95% CI 0.08–0.34), *p* < 0.001) (Figure 3). The majority of these NETLM and EHD patients with liver resection had small bowel as their primary NET site (70%) and peritoneal metastases (PM) as their site of concurrent EHD (93%).

## 4. Discussion

Traditionally, there has been reluctance to operate on NETLM patients in the context of EHD, even when sites of EHD can be completely resected [16,17]. However, this viewpoint has shifted as survival benefit has been demonstrated with appropriate patient selection [18]. In this population-based study, we demonstrate that LDT is associated with prolonged survival in select NETLM patients with concurrent EHD. Within the LDT group, patients undergoing liver resection had improved survival compared to those with nonsurgical LDT.

The decision to operate should be made on a case-by-case basis, and extrahepatic disease should be resected at the time of surgery when possible [19]. Additionally, liver-directed embolization has demonstrated favorable survival advantages even for NETLM patients with substantial EHD who are not surgical candidates [20].

There has been increased interest in pursuing LDT in NETLM patients with extrahepatic involvement. LDT has the potential to provide life-prolonging benefit to NETLM patients with EHD due to the indolent nature of most NETs and the evolving landscape of systemic therapies for advanced NETs [21]. These therapies, which include somatostatin analogs, the tyrosine kinase inhibitor sunitinib, the mammalian target of rapamycin (mTOR) inhibitor everolimus, peptide receptor radionuclide therapy with Lutetium oxodotreotide (^177^Lu DOTA-TATE), and the chemotherapy combination capecitabine and temozolomide (CAPTEM), have been associated with improved disease control and survival [22,23,24,25,26]. As improved systemic treatments have prolonged the life expectancy of metastatic GEPNET patients [27], providers are more willing to purse LDT for patients with advanced disease.

Other studies have similarly demonstrated superior long-term outcomes with surgical debulking in the setting of NETLM with EHD. In a large, single-institution study of 800 NET patients who underwent a total of 1001 cytoreductive operations, the majority of debulkings (84%) included resection of EHD in addition to resection of the primary site with or without liver metastases [28]. The authors associated cytoreductive surgery with low morbidity and prolonged survival, and they support aggressive surgical management of NET patients even in the setting of EHD. An additional single-institution study reported the outcomes of 55 NET patients with EHD who underwent cytoreductive surgery [29]. They reported good hormonal control as well as favorable progression-free survival and OS with surgical debulking.

Despite the increasing number of studies that support cytoreduction of NET EHD, there remains hesitation to perform these surgeries and their benefit is questioned. Our own group has previously demonstrated that NETLM patients with bone metastases may benefit from LDT. Of the 203 GEPNET patients identified with both liver and bone metastases, 14.8% of patients underwent LDT after bone metastasis diagnosis, 22.1% underwent LDT prior to bone metastasis diagnosis, and 63.1% never received LDT. The mOS was significantly longer for patients who received LDT after a diagnosis of bone metastases compared to patients who never received LDT, and it was not significantly different from the mOS of patients who received LDT prior to bone metastasis diagnosis. These findings support that LDT may be associated with improved survival for NETLM patients with bone metastasis [30].

In this current study, we included patients with other sites of concurrent EHD, including the lung, peritoneum, and brain. As in the setting of NETLM with bone metastases, liver involvement is the main determinant of a patient’s prognosis even when other sites of EHD are identified, and LDT may still be warranted [9,31]. Only 22% of NETLM patients with concurrent EHD in our study underwent LDT, and we demonstrated an associated mOS benefit with LDT compared to without (27 vs. 16 months, *p* = 0.006).

Additionally, we focused on patients who underwent liver resection rather than nonsurgical LDT (embolization) as hepatic debulking is considered the preferred management of NETLM when possible [32]. These surgical patients mostly had small bowel NETs (SBNET) and PM as their site of EHD. PMs are reported to occur in up to 20% of patients with SBNETs and have been associated with worse survival [33]. However, there are reports of long-term survivors after surgical debulking for NET patients with PM, and a systemic review of eight prospective studies including 1240 patients who underwent cytoreduction concluded that carefully selected patients may benefit from aggressive surgical resection [34]. In our study, liver resection was associated with longer mOS compared to patients with nonsurgical LDT (138 vs. 13 months, *p* < 0.001).

While our study supports that LDT can be associated with prolonged survival in select NETLM patients with EHD, further research is required to determine which of these patients will derive the most benefit from LDT. This is even more critical when considering patients for surgical resection due to the associated increased morbidity compared to embolization. As neuroendocrine neoplasms are a heterogeneous group of tumors, numerous factors, including differentiation state and tumor grade, primary tumor site, and burden and location of metastatic disease, should be considered when evaluating patients for LDT. For other tumor types with a propensity for hepatic and extrahepatic spread, such as colorectal cancer, risk scores have been developed with the goal of predicting recurrence-free and overall survival [35,36]. The development of a scoring system for NETLM with EHD patients would similarly help with prognostication, and it would ideally reflect disease biology to optimize patient selection for surgical and non-surgical LDT.

As with all population-based database studies, there are inherent limitations in this study. The CCR–OSHPD database is dependent upon provider billing codes, and there may be an under reporting of the presence of EHD. This may have decreased the number of patients reported in this study. Furthermore, the degree of tumor burden and whether EHD was identified prior to surgery or intraoperatively discovered is unknown. A causal relationship between surgical and non-surgical interventions with survival cannot be deduced from our data. Additionally, selection bias may be contributing to the survival outcomes as more fit patients with lower amounts of tumor burden are more likely to be selected for surgical resection rather than embolization. Nevertheless, this study reports real-world practice patterns and supports additional studies to further characterize patient factors that impact outcomes after surgery.

## 5. Conclusions

In this population-based study, LDT, including liver resection and arterial embolization, was associated with increased survival in select patients with NETLM and concurrent EHD. This suggests that the presence of EHD should not preclude LDT with stringent patient selection. Additional studies are warranted to determine the defining characteristics of NETLM patients with EHD who will derive benefit with intervention.

## Figures and Tables

**Table 1:** Frequency of liver metastasis, primary tumor resection, liver resection, liver-directed therapies, and survival by primary tumor site.

Variable ^†^	Gastric *n* = 1045	Pancreas *n* = 1795	Small Bowel *n* = 2071	Colon *n* = 870	Rectum *n* = 3947
Liver Metastasis	77 (7)	713 (40)	309 (15)	215 (25)	120 (3)
Primary NET					
Resection when LM					
present					
No	65 (84)	648 (91)	119 (39)	100 (47)	69 (58)
Yes	12 (16)	64 (9)	189 (61)	115 (53)	51 (42)
Liver resection					
No	72 (94)	660 (93)	242 (78)	200 (93)	112 (93)
Yes	- ^§^	53 (7)	67 (22)	15 (7)	- ^§^
TACE or Y90					
No	60 (78)	585 (82)	236 (76)	182 (85)	82 (68)
Yes	17 (22)	128 (18)	73 (24)	33 (15)	38 (32)
Overall Survival					
[months, median (95% CI)]					
Without LM	NR (139-NR)	52 (45–58)	142 (133–146)	154 (133-NR)	NR (NR-NR)
With LM	18 (9–26)	22 (18–25)	70 (52–80)	9 (7–12)	17 (12–27)

NET, Neuroendocrine tumor; LM, Liver metastases; LDT, liver-directed therapy. ^†^ Variables reported as *n* (%) unless otherwise specified. ^§^—denotes fewer than 10 patients which cannot be reported per CCR Data Use Agreements.

**Table 2:** Patient demographics, clinical characteristics, and survival for all patients with liver metastases and for patients with concurrent liver and extrahepatic metastases grouped by whether they received liver-directed therapy.

Variable ^†^	NETLM *n* = 1434	NETLM and EHD without LDT *n* = 256	NETLM and EHD with LDT *n* = 71	*p*-Value
Age [years, median (range)]	61 (14–92)	62 (33–90)	55 (27–79)	**<0.001**
Sex				0.632
Male	783 (55)	138 (43)	36 (51)	
Race/ethnicity				0.653
White	634 (67)	109 (68)	47 (75)	
Black	85 (9)	- ^§^	-	
Hispanic	153 (16)	26 (16)	-	
Asian/Pacific Islander	77 (8)	14 (9)	-	
Comorbidities				0.265
None	1006 (70)	129 (62)	48 (73)	
One	276 (19)	48 (23)	11 (17)	
≥Two	152 (11)	32 (15)	-	
Primary NET site				0.109
Stomach	1353 (11)	13 (5)	-	
Pancreas	2221 (18)	109 (43)	20 (28)	
Small Bowel	2498 (20)	53 (21)	24 (34)	
Colon	1068 (9)	52 (20)	13 (18)	
Rectum	5090 (42)	29 (11)	-	
Primary NET resection				0.112
No	1001 (70)	167 (65)	39 (55)	
Yes	431 (30)	89 (35)	32 (45)	
Liver-directed therapy modality				
Liver resection	148 (10)		23 (32)	
TACE	270 (19)		49 (69)	
Y90	54 (4)		-	
Concurrent EHD site ^‡^				
Any	327 (23)			
Lung	91 (28)	78 (30)	13 (18)	**0.043**
Peritoneum	159 (49)	113 (44)	46 (65)	**0.002**
Bone	120 (37)	101 (39)	19 (27)	0.050
Brain	19 (6)	18 (7)	-	0.073
Number of EHD sites ^‡^				0.424
0	1107 (77)			
1	274 (19)	210 (82)	64 (90)	
≥2	53 (4)	46 (18)	-	
Any chemotherapy				**<0.001**
No	830 (58)	159 (62)	28 (39)	
Yes	565 (39)	95 (37)	38 (54)	
Overall Survival				**0.006**
[months, median (95% CI)]	26 (7–72)	16 (6–52)	27 (7–83)	

NETLM, Neuroendocrine tumor liver metastases; EHD, extrahepatic disease; LDT, liver-directed therapy; TACE, trans-arterial chemoembolization; Y90, yttrium-90 radioembolization. ^†^ Variables reported as n (%) unless otherwise specified. ^‡^ Total not equal to 100% when multiple sites of EHD present ^§^—denotes fewer than 10 patients which cannot be reported per CCR Data Use Agreements. Bold values denote statistical significance at the *p* < 0.05 level.

## Data Availability

Data are available from the California Cancer Registry, found at “https://www.ccrcal.org/retrieve-data/data-for-researchers/how-to-request-ccr-data/, accessed on 16 September 2022”.
